# Self-Adjuvanting Bacterial Vectors Expressing Pre-Erythrocytic Antigens Induce Sterile Protection Against Malaria

**DOI:** 10.3389/fimmu.2013.00176

**Published:** 2013-07-04

**Authors:** Elke S. Bergmann-Leitner, Heather Hosie, Jessica Trichilo, Elizabeth DeRiso, Ryan T. Ranallo, Timothy Alefantis, Tatyana Savranskaya, Paul Grewal, Christian F. Ockenhouse, Malabi M. Venkatesan, Vito G. DelVecchio, Evelina Angov

**Affiliations:** ^1^Malaria Vaccine Branch, Walter Reed Army Institute of Research, Silver Spring, MD, USA; ^2^Vital Probes, Inc., Mayfield, PA, USA; ^3^Division of Bacterial and Rickettsial Diseases, WRAIR, Silver Spring, MD, USA; ^4^Division of Entomology, WRAIR, Silver Spring, MD, USA

**Keywords:** *E. coli*, *Shigella*, malaria, self-adjuvanting, cellular targeting, bacterial vaccine vector, CelTOS, CSP

## Abstract

Genetically inactivated, Gram-negative bacteria that express malaria vaccine candidates represent a promising novel self-adjuvanting vaccine approach. Antigens expressed on particulate bacterial carriers not only target directly to antigen-presenting cells but also provide a strong danger signal thus circumventing the requirement for potent extraneous adjuvants. *E. coli* expressing malarial antigens resulted in the induction of either Th1 or Th2 biased responses that were dependent on both antigen and sub-cellular localization. Some of these constructs induced higher quality humoral responses compared to recombinant protein and most importantly they were able to induce sterile protection against sporozoite challenge in a murine model of malaria. In light of these encouraging results, two major *Plasmodium falciparum* pre-erythrocytic malaria vaccine targets, the Cell-Traversal protein for Ookinetes and Sporozoites (CelTOS) fused to the Maltose-binding protein in the periplasmic space and the Circumsporozoite Protein (CSP) fused to the Outer membrane (OM) protein A in the OM were expressed in a clinically relevant, attenuated *Shigella* strain (*Shigella flexneri* 2a). This type of live-attenuated vector has previously undergone clinical investigations as a vaccine against shigellosis. Using this novel delivery platform for malaria, we find that vaccination with the whole-organism represents an effective vaccination alternative that induces protective efficacy against sporozoite challenge. *Shigella* GeMI-Vax expressing malaria targets warrant further evaluation to determine their full potential as a dual disease, multivalent, self-adjuvanting vaccine system, against both shigellosis, and malaria.

## Introduction

While traditional whole-cell, killed, or live-attenuated microorganism-based vaccines have been the most effective for disease prevention, a large number of subunit vaccines are currently in development and are being evaluated for their ability to elicit protective immune responses. These approaches include DNA vaccines, recombinant protein (with adjuvant or as conjugates), viral vectors, and bacteria expressing full-length or immunologically crucial antigens (1–11). Vaccine development for the three major human diseases, namely HIV, tuberculosis, and malaria, has been primarily impeded by host immune evasion mechanisms employed by pathogens, the poor immunogenicity of protective antigens and/or the inability to generate effective whole-organism vaccines. Poor immunogenicity can often be overcome by administering the vaccine with potent adjuvants (12, 13). However, the limited repertoire and availability of human-use adjuvants is hampering the clinical vaccine development pipeline (14). Using recombinant viruses or bacteria as vectors could overcome some of these limitations. Vaccination with live-attenuated organisms mimic the immune stimulation of natural infections and offer induction of a more natural and robust immune response. Although some concerns over their safety have prevented their universal acceptance and licensure; recent advances toward increased safety through detoxification and unique delivery routes may quell some of these concerns. Using bacteria as recombinant vectors to mount an immune response against xenogeneic transgenes has several advantages: (1) pathogen-associated molecular patterns (PAMPs) on the bacteria are recognized by specialized pattern recognition receptors (PRRs) of the host and lead to the activation of strong innate immune responses (15). This is the crucial first step in initiating an antigen-specific adaptive immune response. Depending on the type of PAMPs, distinctive types of adaptive immune responses are induced. (2) Complement factors MBL, C1q, and C3b also recognize PAMPs which results in proteolytic activation of the complement cascade. Binding with complement mediates the targeting of the bacteria to the complement receptors (CR) on antigen-presenting cells (APC) (16) therefore improving the antigen presentation of xenogeneic antigens. (3) In individuals with pre-existing immunity to the bacterium, cross-linking between antigen receptor and CR results in strong activation of the respective B cells (17, 18) thus improving humoral immune responses. (4) Opsonized pathogens targeted to CR on follicular dendritic cells ensure long-lasting immunity (19–21). (5) Finally, using bacteria of clinical relevance as vectors for delivery of xenogeneic pathogen-derived antigens holds the potential for their application as dual-use vaccines. Currently, several virulence-attenuated bacteria are used as vaccines and are either commercially available or in clinical development, i.e., *Salmonella enterica* serovar Typhi Ty21a (22, 23), *Vibrio cholera* CVD 103-HgR (24, 25), *Mycobacterium bovis* BCG (26–28), *Shigella dysenteriae* Type 1, *Shigella flexneri* 2a, and *Shigella sonnei* (29–33).

These advantages warrant further evaluation of recombinant bacteria as vectors for delivering heterologous target antigens either by co-expression, adsorption, or encapsulation (28, 34–37). Traditionally, microorganisms have been inactivated or killed using methods with strong denaturing conditions involving heat or chemical treatments such as formaldehyde or formalin. This process is meant to ensure the safety of the formulation but the harsh treatment can negatively affect the structure of the pathogen’s proteins and thus antigenicity of key protective antigens (38, 39). Molecular methods to sustain surface antigen functionality and integrity that circumvent these denaturing conditions include the controlled expression of PhiX174 gene E leading to the concept of Bacterial Ghosts (BGs) as a vaccine delivery platform (40, 41). A new approach to inactive bacteria not previously described uses genetic means to express inhibitors of key metabolic processes that disrupt cellular functions without significantly altering bacterial cell structure integrity. In the current study, we utilize this Gene-Mediated-Inactivation Vaccine (GeMI-Vax) process to generate inactivated Gram-negative bacteria carrying heterologous protein antigens. In GeMI-Vax production, a Gram-negative pathogen is transformed with plasmids containing a gene for an antigen of interest and the GeMI-Vax inactivation gene, ColE3, which encodes a colicin that degrades mRNA. GeMI-Vax bacteria serve as the antigen delivery system in the context of whole bacterial cells that are rendered non-replicating and non-viable through this type of genetic manipulation. Moreover, since these bacteria are not chemically modified, conformational epitopes on the recombinant antigens, and the bacterial derived PAMPs (such as lipopolysaccharide, lipoproteins, flagellin, and DNA) are unchanged allowing for the induction of potent immune responses. The advantage of using GeMI-Vax bacteria as delivery platform compared to traditional adjuvants is that a wide range of PAMPs can trigger distinct PRRs, both surface bound (e.g., TLR-4) and intracellular (e.g., TLR-9) thus resulting in the engagement of multiple signaling pathways.

Malaria caused by *Plasmodium falciparum* results in serious illness and often leads to death if left untreated. The development of an efficacious vaccine to prevent this global disease is of utmost importance. There is an urgent need to develop a highly efficacious, low cost, self-adjuvanting, pre-erythrocytic stage malaria vaccine from *P. falciparum* target antigens (sporozoite and liver stages) to protect populations in malaria endemic regions. In initial studies, *Escherichia coli* GeMI-Vax were co-transformed with plasmids expressing the malaria target antigen and the bacterial host inactivation gene product. The malaria targets used in the *E. coli* experiments was the rodent malaria *Plasmodium berghei* Circumsporozoite Protein (*Pb*CSP) and the Cell-traversal protein of ookinetes and sporozoites (*Pb*CelTOS). Three sub-cellular localizations of the target were evaluated; expression in the cytosol (Cyto), the periplasmic space (PPS), and the outer membrane (OM) of the bacterial carrier. Localization to the PPS and OM was accomplished through genetic fusions between the Maltose-Binding Protein (MBP) and the Outer Membrane Protein A (OmpA), forming chimeric proteins with the malaria targets. Culture conditions were optimized to maximize antigen expression, effective bacterial inactivation, and the isolation of whole, intact GeMI-Vax cells. Since no harsh physical or chemical agents are employed, proteins, and other immune-stimulating components of GeMI-Vax cells are intact and recognized by the immune system in the same manner as their live pathogenic counterpart. Thus they elicit a robust immune response without the need for additional adjuvants. GeMI-Vax cells were evaluated for their ability to induce protection against malaria challenge in murine challenge models, and both the humoral and cellular immune responses were evaluated.

Evidence of immunological potency and protection from studies performed using *E. coli* GeMI-Vax supported translation to the clinically more relevant whole-cell *Shigella flexneri* as the delivery platform. The GeMI-Vax platform was used to inactivate engineered *Shigella flexneri* 2a (15G strain) expressing *P. falciparum* CelTOS (*Pf*CelTOS) in the PPS or *P. falciparum* CSP (*Pf*CSP) on the OM. Similar to *E. coli* GeMI-Vax, the *Shigella* GeMI-Vax cells have none of the disadvantages of chemical killing or regulatory hurdles associated with live vaccines. The live-attenuated *S. flexneri* used in this study were previously shown to be safe and protective in an animal model when administered intranasally (42). Both applications of GeMI-Vax, *E. coli*, and *S. flexneri* 2a (15G strain), expressing malaria targets on the surface of bacteria elicited antigen-specific antibodies and IFN-γ producing T cells when expressed on the OMs. Malaria antigen localized to intracellular spaces, i.e., periplasmic and cytosol, skewed toward cellular responses with no significant levels of antibodies detected. Thus the protective efficacy of the different constructs supports GeMI-Vax as a vaccine vector system for delivery of target antigens. Additionally, expressing target antigens at different sites on bacteria influences the type of immune responses induced and allows for investigations into antigen-specific immune correlates of protection.

## Materials and Methods

### PbCSP in the cytosol for *E. coli* expression

The *Plasmodium berghei* (*Pb*) CSP gene coding sequence was obtained from the plasmid pET(K-) PbCSP-tr. The gene insert was synthesized by Retrogen Inc. (San Diego, CA, USA) using a WRAIR proprietary codon harmonized nucleotide sequence for optimal expression in *E. coli* (43). The *Pb*CSP nucleotide sequence was truncated in the total number of repeat motifs for cloning purposes. The gene product was 572 bp long and contained a His tag at the 5′end. The final expressed product has a molecular weight of 21 kDa, but runs anomalously on gradient Tris-Glycine SDS-PAGE (Invitrogen, Carlsbad, CA, USA) due to the complex nature of its structure. The *Pb*CSP-tr DNA from pET(K-)*Pb*CSP-tr was used for constructing the OM-*Pb*CSP and PPS-*Pb*CSP GeMI-Vax plasmids as well as for expressing *Pb*CSP in the cytosol of *E. coli* (Cyto-*Pb*CSP).

### PbCSP in the outer membrane for *E. coli* expression

The *Pb*CSP gene from pET(K-)*Pb*CSP-tr was PCR amplified and cloned into the pVPIHS64K OM vector yielding an *E. coli* OmpA-*Pb*CSP fusion protein. After the sequence was confirmed, the plasmid was used to transform NM522 *E. coli* cells. Positive clones were identified by colony PCR using vector specific primers. Expression was confirmed by induction of bacterial cultures with 1 mM isopropyl-thio-β-galactoside (IPTG) (Roche, Indianapolis, IN, USA), and analyzed by whole-cell extraction and SDS-PAGE Western blotting (Invitrogen) using an anti-His6 antibody (Sigma-Aldrich, St. Louis, MO, USA). Expression was detected at the expected molecular weight of 60 kDa for the fusion protein. The *Pb*CelTOS codon harmonized nucleotide sequence was similarly constructed to be expressed in the OM and the PPS of *E. coli* (44). The details for growth, expression, and isolation are identical to those used for the *Pb*CSP GeMI-Vax constructs.

### PbCSP in the periplasmic space for *E. coli* expression

The *Pb*CSP gene coding sequence was obtained from plasmid pET(K-)PbCSP-tr. The gene was PCR amplified and cloned into the p2XK MBP vector, yielding an *E. coli* MBP-*Pb*CSP fusion protein. After the sequence was confirmed, the plasmid was used to transform NM522 cells. Positive clones were identified by colony PCR using vector specific primers. Expression was confirmed by induction of bacterial cultures with 1 mM IPTG; and analyzed by whole-cell extraction and SDS-PAGE Western blotting using an anti-His6 antibody. Expression was detected at the expected molecular weight of 64 kDa for the fusion protein.

### Preparation of *E. coli* GeMI-Vax cells

*E. coli* NM522 cells were grown to an OD_600_ of 0.3 at 30°C in Hyperbroth (Accurate Chemicals & Scientific Corp., Westbury, NY, USA) with 2% Glucose Nutrient Mixture™ (Accurate Chemicals & Scientific Corp.) media containing 40 μg/mL kanamycin (Sigma-Aldrich) and 20 μg/mL gentamicin (Sigma-Aldrich). Key steps in the GeMI-Vax expression and isolation are outlined in Table 1. Briefly, *E. coli* cells were induced with 1 mM IPTG to express the malaria target antigen for 1 h. Induction of *colE3* gene, which encodes a colicin that degrades mRNA, was used for cell inactivation. The *colE3* inactivation gene was cloned behind the araBAD promoter in a pBAD vector (Invitrogen) (modified for gentamicin selection) using *Afl*II and *Nco*I restriction sites and expression was induced by the addition of 2.5% l-(+)-arabinose (≥99%, Sigma-Aldrich) for 1.5 h. During cell inactivation, 100 μg/mL gentamicin was added to guarantee plasmid retention. As a final step to ensure 100% cell inactivation, 2 mg/mL solid streptomycin (Sigma-Aldrich) was added to cultures for 2 h. Cells were collected by centrifugation at 5,000 rpm for 15 min, pellets were resuspended in 50 mL sterile water, and the suspension was stored with 800 μg/mL of streptomycin overnight at 4°C. To determine final cell suspension sterility, 100 μL of cells were plated onto antibiotic-free Luria Broth agar plates and also inoculated into 10 mL liquid culture media with no antibiotics. LB plates and culture tubes were incubated at 37°C overnight or for 37°C for 5 days, respectively.

**Table 1:** **Summary steps for production of GeMI-Vax cells**.

Time (min)	OD_600_	Temperature		Induction		Description
		*E. coli* (°C)	*Shigella* (°C)	*E. coli*	*Shigella*	
0	0.3	30	30	IPTG	IPTG	Induce malaria Ag expression
60	0.6	30	40	Arabinose	Temp shift	Induction inactivation gene expression
150		30	40	–	–	Final inactivation with streptomycin
270		30	40	–	–	Harvest and wash cells
48 h		30	40	–	–	Lyophilization

### Washing and lyophilization

For final storage, the cell pellets were washed extensively by sequential centrifugation and resuspension with four exchanges of sterile water to remove any residual antibiotics. After the final wash, cells were aliquoted into cryotubes, snap-frozen in liquid nitrogen and kept at −80°C overnight. Frozen cells were lyophilized using Labconco-Fast Freeze Flasks (Labconco, Kansas City, MO, USA) and a Flexi-Dry™ Microprocessor (μP) Control-Corrosion Resistant Freeze Dryer (FTF Systems, Stone Ridge, NY, USA). Lyophilization was completed over a two day period at a temperature of −70°C under vacuum (600 mTorr). The final lyophilized vials were stored at 4°C.

### PfCelTOS in the periplasmic space for *Shigella* expression

A plasmid containing the *Plasmodium falciparum* (*Pf*) CelTOS gene served as the template for PCR amplification. Briefly, the parent plasmid included a 522 bp DNA fragment of *Pf*CelTOS 3D7 designed by using the codon-harmonization algorithm (43, 45) and cloned into a modified pET(K−) expression vector (Novagen, Madison, WI, USA). Oligonucleotides were designed for cloning the *Pf*CelTOS sequence into the p2xK periplasmic vector yielding a fusion protein with the MBP. After sequence confirmation, the plasmid was transformed into *S. flexneri 2a* (15G *strain*) (ATCC, Manassas, VA, USA). Positive clones were identified by colony PCR using vector specific primers. Expression testing was accomplished by induction of bacterial cultures with IPTG followed by whole-cell extraction and SDS-PAGE Western blot using an anti-MBP antibody (New England BioLabs, Ipswich, MA, USA). Expression was confirmed at the expected molecular weight of 61 kDa for the fusion protein.

### PfCSP in the outer membrane for *Shigella* expression

The *Plasmodium falciparum* (*Pf*) CSP gene coding sequence was synthesized by DNA 2.0 (Menlo Park, CA, USA). DNA 2.0 adjusted the coding sequence for optimized expression in *E. coli*. The gene was 807 bp long and a His tag was added to the 5′end. The highly repetitive central repeat region was retained in its entirety. The gene was PCR amplified and cloned into the pVPIHS64K OM vector, yielding an *E. coli* OmpA-*Pf*CSP fusion protein. After the sequence was confirmed, the plasmid was used to transform *S. flexneri 2a* (15G *strain*). Positive clones were identified by colony PCR using vector specific primers. Expression testing was performed by induction of bacterial cultures with IPTG followed by whole-cell extraction and SDS-PAGE Western blot using an anti-His6 antibody (Sigma). Expression was detected at the expected molecular weight of 68.5 kDa.

### Construction of pλColE3 plasmid

A plasmid synthesized by DNA2.0 (Menlo Park) designated GV001λVPI was used to engineer the GeMI-Vax inactivation gene ColE3 behind the lambda promoter. This plasmid contained the ColE3 gene under control of the araBAD promoter (ColE3 cassette), a malaria gene under control of the λ promoter (a temperature sensitive promoter), the gentamicin resistance marker, and the pUC origin of replication and was used as the template for constructing the pλColE3 plasmid. The ColE3 cassette was removed by digesting the plasmid with *Pme*I and *Sfi*I. After gel purification the vector backbone was self ligated and transformed into *E. coli* and plated onto gentamicin plates. DNA plasmid purification was performed. The malaria gene was removed by digesting with *Nde*I and *Not*I and the backbone vector was gel purifying. The ColE3 gene was amplified by PCR using primers that incorporated *Nde*I and *Not*I recognition sites. The amplicon was digested and then ligated behind the λ promoter, in place of the malaria gene. The newly constructed “GV200” plasmid was transformed into *E. coli*. Sequence confirmation was performed at Genewiz Inc (South Plainfield, NJ). Expression of the ColE3 gene was also confirmed by expression profile.

### Co-expression of malaria target and inactivation genes on same plasmid for *Shigella* expression

A single plasmid was created which contained either of the malaria antigens as well as the GeMI-Vax inactivation gene, ColE3. The malaria antigens were controlled by the IPTG inducible pTac promoter as described above. The ColE3 inactivation gene was controlled by the lambda promoter, also described above. The malaria antigen cassette, consisting of the lac repressor, the pTac promoter, the malaria antigen, and the terminator, was amplified by PCR using primers which contained restriction enzyme tails. Once amplification was confirmed by agarose gel electrophoresis, restriction enzyme digestion was performed. The cassette was then ligated into the GV200 vector, which contained the GeMI-Vax inactivation gene ColE3 under the control of the lambda promoter, which was then linearized with the same restriction enzymes. Each of the malaria target antigens was then subcloned into the GV200 vector described above resulting in the double target GV200-p2xk*Pf*CelTOS and GV200-pHS64*Pf*CSP plasmids, respectively. Positive clones were obtained and the inserted sequences were verified by DNA sequencing. Cell inactivation for these constructs was achieved by a temperature shift from 30 to 40°C resulting in the denaturation of the c1857 repressor and induction of expression of the inactivation gene. Upon sequence confirmation the plasmids were transformed into *Shigella* and antigen expression and inactivation studies were performed.

### Preparation of *S. flexneri* 15G-GeMI-Vax cells

*S. flexneri* 15G is an *asd* mutant strain derived from the parent 2457T strain that is non-replicating in LB medium in the absence of added diaminopimelic acid (DAP) or within eukaryotic cells that lack an endogenous source of DAP. Bacteria cells that are *asd* mutants have an obligate requirement for DAP, an essential constituent of the cell wall. APF Luria Broth (Athena ES, Baltimore, MD, USA) supplemented with 2% glucose, 20 μg/mL gentamicin, 100 μg/mL DAP, was inoculated with 0.15 OD_595_ of stationary phase culture *S. flexneri* 2a 15G. The cultures were grown at 30°C with shaking at 250 RPM to an OD_595_ of 0.3 and induced with 1 mM of IPTG. One hour following IPTG induction, an additional 20 μg/mL of gentamicin was added and the temperature was raised to 40°C for an additional 90 min to induce ColE3 induction and cell inactivation. 100 μg/mL of streptomycin was added after the 90 min incubation and the cells continued to incubate for an additional 2.5 h. Cells were collected by centrifugation at 4,000 rpm for 20 min at 4°C. Pellets were resuspended in cold, sterile 1 x PBS (Quality Biological, Gaithersburg, MD, USA). This step was repeated several times. After a fourth centrifugation, the pellet was resuspended in 1 × PBS with 100 μg/mL of streptomycin and incubated with gentle rocking at 4°C overnight. Streptomycin was removed by washing the cell pellet with 1 × PBS with at least four exchanges. Antigen expression was monitored by whole-cell extraction, SDS-PAGE, and Western blot.

### Washing and lyophilization

*Shigella* GeMI-Vax cell pellets were washed extensively by the process of sequential centrifugation and resuspension with four exchanges of sterile 1 × PBS to remove any residual antibiotics. All subsequent steps are identical to those described above.

### Immunizations

Mice used for immunizations were either 6- to 8-week-old BALB/c-J or C57BL6 (Jackson Laboratories, Bar Harbor, ME, USA) female mice (*n* = 10 for challenges and *n* = 5 for cellular assays). For all *Pb*CSP and *Pb*CelTOS based immunization, BALB/c were immunized three times in the scruff of the neck at 3 week intervals subcutaneously (sc) with either 200 μL of 1.7 × 10^9^ GeMI-Vax cells or 1 μg r*Pb*CSP-tr/ISA 720 (Seppic, Inc.). For *Pf*CelTOS-based immunizations, BALB/C were immunized three times at 3 week intervals in the scruff of the neck subcutaneously (sc) with either 1.7 × 10^9^ GeMI-Vax cells delivered in two 100 μL aliquots or with 10 μg r*Pf*CelTOS/ISA 720 (Seppic, Inc.). All recombinant proteins used had endotoxin levels below the limits of detection (Pyrochrome LAL Kit, Cape Cod Associates, MA, USA). For *Pf*CSP based immunizations, C57BL6 mice were immunized four times with 1.7 × 10^9^ GeMI-Vax cells delivered in two 100 μL aliquots in the scruff of the neck. Sera were collected one day prior to each immunization and four weeks after the last immunization, prior to challenge with either live wild-type *P. berghei* sporozoites or live *P. berghei P. falciparum* CSP transgenic sporozoites (46).

### Challenge

Thirty days after the final immunization, mice were challenged with either wild-type *Plasmodium berghei* sporozoites dissected from infected mosquito salivary glands (4,000 sporozoites for BALB/c) by subcutaneous inoculation (into the inguinal region) (47) or (5,000 *Pb*-*Pf*CSP-transgenic sporozoites for C57BL6) by intravenous inoculation. Infection was determined by the presence of blood stage parasites in Giemsa-stained thin blood smears on days 6, 8, 10, and 14 after challenge. Mice that were aparasitemic on day 14 were scored as protected (i.e., sterile protection).

### ELISA

The ELISA was performed as previously described (45, 48). Briefly, 96-well plates (Immunolon 2 HB, Thermo Milford, MA, USA) were coated by overnight incubation with either 50 μL of r*Pb*CSP-tr at 0.05 μg or 100 μL *Pf*CelTOS and *Pf*CSP at 0.025 and 0.035 μg per well, respectively, in 1 × PBS at 4°C. Sera were serially diluted in blocking buffer and incubated at 37°C for 2 h. Antibody concentration was determined by establishing a standard curve (run with each assay) with purified mouse IgG. For each serum, we determined a concentration that was within the linear portion of the reaction curve and used this dilution to extrapolate the actual antibody concentration in the assay wells. Antibody avidity measurements were done using the chaotropic agent sodium isothiocyanate (NaSCN) as previously described (48). Briefly, antigen-specific interactions were disrupted by the addition of varying amounts of the chaotropic agent NaSCN. Results are reported as the effective concentration of NaSCN required to release 50% of antisera (ED_50_) and are used to evaluate antibody avidities for the different vaccination groups.

### Immunofluoresence assay

Immunofluoresence assay (IFA) were performed as previously described using salivary gland *P. berghei* or *P. falciparum* sporozoites (45).

### ELISpot assays

Cells were stimulated with various concentrations of recombinant protein or 1 μg/mL *P. berghei* CSP peptides CS57-70 (49), CS58-65 (50), CS260-279 (51), and CS252-260 (52). IFN-γ or IL-4 specific ELISpot assays (R&D Systems, Minneapolis, MN, USA) were performed as previously described (45).

### Western blot analysis

Protein expression was determined by separating whole-cell extracts of GeMI-Vax cells (OD_600_ = 0.3) on 4–20% Tris-Glycine SDS-PAGE. Proteins were transferred to 0.2 μm nitrocellulose membranes (Invitrogen) and blocked with 1 × PBS pH 7.4, 0.1% Tween 20. Blots were probed with *Pb*CSP-specific mAb 4B10 (generously provided by Dr. Robert Wirtz, CDC), or *Pf*CSP-specific mAbs (clones WR3 and WR7, WRAIR, Malaria Vaccine Branch) or *Pf*CelTOS-specific mAbs (clones 3D11.E2, 3C3.C2) or Ag-specific polyclonal antibodies. Membranes were washed after 1 h incubation and then incubated with alkaline-phosphatase-conjugated secondary antibodies (Promega, Madison, WI, USA), with either goat-anti-mouse IgG (1:10,000), goat-anti-human IgG (1:10,000), or goat-anti-rabbit IgG (1:20,000). Blots were washed and then developed for 10 min at RT with NBT/BCIP in 0.1 M NaCl, 5 mM MgCl_2_, and 0.1 M Tris-HCl pH 9.0. For detection of *Pb*CSP, membranes were probed with HRP-conjugated mouse mAb 4B10 (1:20,000) for 2 h. Membranes were washed with 1 × PBS, 0.05% Tween 20 prior to applying the Bio-Rad Immun-Star™ WesternC™ reagent (Bio-Rad, Laboratories, Hercules, CA, USA). Bio-Rad’s VersaDoc Imaging system was used to measure the band intensity after 60 s exposures.

### Statistical analysis

The protective effect of vaccination against *P. berghei* sporozoite challenge was evaluated using the Fisher’s exact test comparing differences between the GeMI-Vax expressing malaria antigens and empty cells; adjuvant control groups and recombinant protein vaccine groups, respectively. Statistical significance of the serological data and cellular responses was tested using ANOVA and Student *T*-tests (two-sided), respectively, employing the SigmaPlot v12 (Systat Software, Inc., San Jose, CA, USA). Data not meeting normality testing was subsequently tested using Kruskal–Wallace One-way analysis of variance on ranks.

## Results

### *E. coli* gene-mediated-inactivation vaccine expressing malaria antigens

Gram-negative *E. coli* were selected as the bacterial vector system to evaluate the GeMI-Vax approach for malaria. These studies were designed to investigate: (1) the relationship between antigen-specific dosage levels, (2) effect of sub-cellular localization of antigen, (3) the type of immune responses induced, and (4) the efficacy of the GeMI-Vax vaccine platform in *E. coli*. Prior to embarking on immunological studies, bacterial growth, expression, localization, and inactivation conditions were established.

Malaria target sequences used for co-expression were codon harmonized for optimal expression and protein folding in the bacterial host. The approach relies on codon frequency matching between the antigen-“donor” species (in this case *Plasmodium*) and the “recipient” species (in this case *E. coli*) yielding regulated co-translational folding (43).

**Figure 1:**
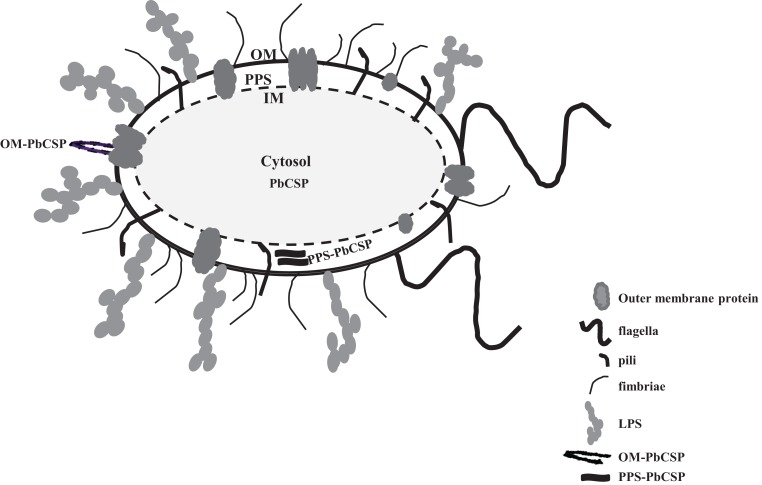
**Diagram of Gram-negative bacterium representing the outer membrane, periplasmic space, and the cytosol**. *Pb*CSP expression is represented as either a fusion with outer membrane protein OmpA or in the periplasmic space with MBP Inner membrane (IM). Cartoons of various cell surface molecules are included but not drawn to scale, for instance pili, flagella, LPS, and transmembrane proteins.

**Figure 2:**
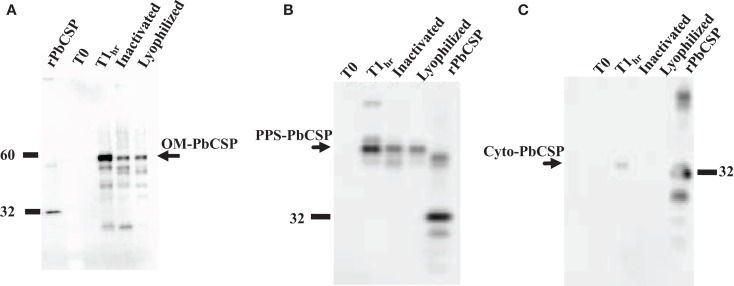
**Western blot expression profiles of GeMI-Vax *Pb*CSP from different sub-cellular localizations**. **(A)** Targeting *Pb*CSP to the outer membrane, OM-*Pb*CSP. Lanes: 5 ng r*Pb*CSP; un-induced cells (T0), 1 h post IPTG induction (T1h), post ColE3 inactivation, post lyophilization; **(B)** Targeting *Pb*CSP to the periplasmic space, PPS-*Pb*CSP. Lanes: un-induced cells (T0), 1 h post IPTG induction (T1h), post ColE3 inactivation, post lyophilization; 1 μg r*Pb*CSP. **(C)** Expression of *Pb*CSP to the cytoplasmic space. Lanes: un-induced cells (T0), 1 h post IPTG induction (T1h), post ColE3 inactivation, post lyophilization, 1 μg r*Pb*CSP. Arrow points to r*Pb*CSP which migrates at ∼32 kDa. Final lyophilized product loaded at 1.2 × 10^7^ cells per lane.

**Figure 3:**
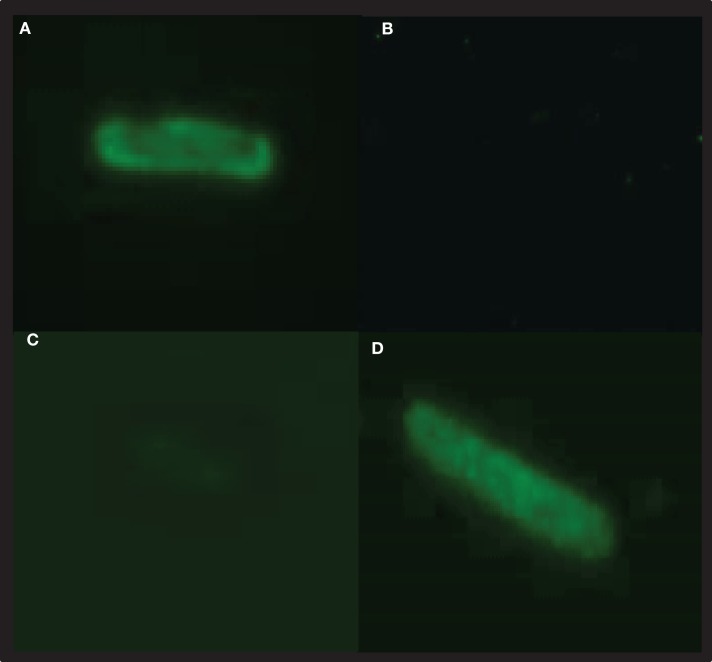
***Pb*CSP-specific antibodies react with recombinant expressing *Pb*CSP GeMI-Vax bacteria**. Bacteria expressing *Pb*CSP in the outer membrane **(A,B)** or periplasmic space **(C,D)** were stained with the *Pb*CSP-specific mAb 4B10 **(A,C,D)** or control mAb (2C6.7 MSP1_19_ specific mAb) **(B)**. Bacteria were stained for surface expression **(A,C)** or after permeabilization for intracellular expression **(D)**. Representative images were taken at 1,000× magnification.

Malaria targets such as *Plasmodium* CSP are characterized by a central, highly repetitive region, which contains varying numbers of repeat motifs. These repeat motifs have made it technically difficult to synthesize full-length CSP genes. Thus, for this purpose, a reduced number of central repeats were incorporated into the *Pb*CSP sequence. Several plasmid constructs were generated to express *Pb*CSP on the OM, to the PPS or in the cytosol of recombinant bacteria (Figure 1). Fusing *pbcsp* with the *ompa* gene resulted in insertion into the OM while fusing the CSP gene with the MBP gene yielded expression to the PPS. Removal of the MBP fusion partner from the latter construct resulted in the expression of CSP to the cytosol (Cyto). Despite the engineering to truncate the central repeat region and expression as fusion proteins, expressed CS proteins were recognized by *Pb*CSP-repeat specific monoclonal antibody 4B10 (Figure 2) and by *Pb*CSP-specific polyclonal antibodies induced in mice with recombinant *Pb*CSP/Montanide ISA 720 (data not shown). These results confirm the cellular expression and immune-recognition of the CS proteins. High levels of heterologous expression were measured in the OM (Figure 2A) and the PPS (Figure 2B), while cytosolic expression was only detectable after IPTG induction and lost after inactivation and lyophilization (Figure 2C). The cytosolic expression likely resulted in intracellular proteolysis in the absence of a fusion partner. Immunostaining of *E. coli* GeMI-Vax using a *Pb*CSP-specific mAb verified the localization and distribution of fusion proteins (Figure 3). Staining of unfixed OM-*Pb*CSP showed a circumferential staining pattern consistent with its surface expression through fusion with the OM protein (Figure 3A); while surface staining of the unfixed PPS-*Pb*CSP cells was negative (Figure 3C) and required permeabilization of the cells to detect the intracellularly expressed PPS-*Pb*CSP (Figure 3D). PPS-*Pb*CSP staining in the PPS appeared uniform throughout the bacterium. No surface staining was observed of OM-*Pb*CSP GeMI-Vax probed with an irrelevant mAb (Figure 3B).

### CSP expression to different cellular compartments on *E. coli* dictates immune responses

**Figure 4:**
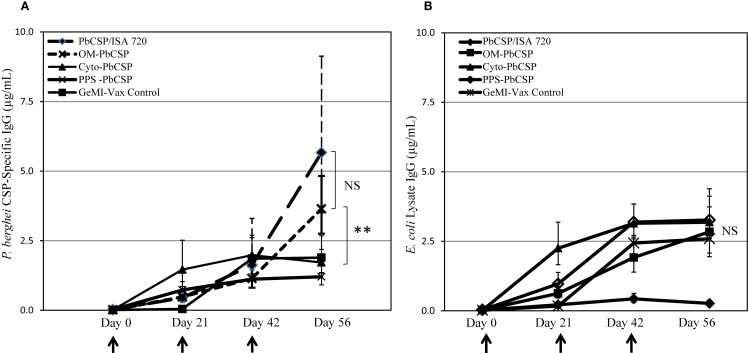
**OM-*Pb*CSP GeMI-Vax bacteria induce strong humoral immune responses**. Sera from mice immunized with either OM-*Pb*CSP, PPS-*Pb*CSP, Cyto-*Pb*CSP, or recombinant *Pb*CSP adjuvanted with Montanide ISA-720 were collected two days prior to each immunization and tested for reactivity against r*Pb*CSP **(A)** or on *E. coli* lysates **(B)**. Data are expressed as geometric mean in μg/mL of antigen-specific IgG (*n* = 15 mice/group), and the error bars indicate the 95% confidence interval. NS denotes not statistically significant. Asterisk indicates statistical significance, **p < 0.001; Kruskal–Wallace One-Way Analysis of Variance on Ranks.

**Figure 5:**
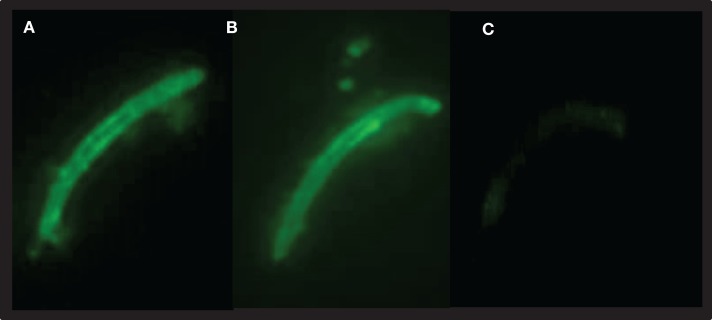
**OM-*Pb*CSP-GeMI-Vax (A) and *Pb*CSP/ISA-720 (B) induce antibodies that react with *P. berghei* sporozoites by indirect immunofluorescence**. The reactivity was specific as sera from mice immunized with empty GeMI-Vax did not react with sporozoites **(C)**. Representative images were taken at 1,000 × magnification, staining at 1:10,000 dilutions.

To evaluate the immune potential of bacterial vectors to induce malaria-specific responses, mice were immunized with *E. coli* GeMI-Vax cells. Based on protein expression estimates made from Western blots, the total antigen doses delivered were 0.5 μg/dose for OM-*Pb*CSP, 0.1 μg/dose for the PPS-*Pb*CSP and below detection limit for the Cyto-*Pb*CSP. The number of cells delivered was based on experiments performed to determine tolerability in BALB/c mice, thus the maximum number of cells delivered for all constructs was 1.7 × 10^9^ cells/dose. OM-*Pb*CSP immunized mice and a reference group (mice immunized with recombinant 1 μg *Pb*CSP/Montanide ISA-720) showed the highest titers of *Pb*CSP-specific antibodies (Figure 4A). However, in all cases, the OM-*Pbf* CSP induced CSP-specific antibody responses that were significantly greater than for any other GeMI-Vax group (Kruskal–Wallace One-way analysis of variance on ranks, *p* < 0.001). PPS- and Cyto-*Pb*CSP immunizations induced responses that could not be boosted and were not distinguishable from background, “empty” GeMI-Vax cells. Antibodies to *E. coli* were measured against an *E. coli* lysate by ELISA and did not vary by GeMI-Vax groups (Figure 4B). For GeMI-Vax groups, only the third immunization with OM-*Pb*CSP boosted *Pb*CSP-specific responses, however they were not statistically different compared with responses induce by the recombinant protein *Pb*CSP/ISA 720 on Day 56, just prior to sporozoite challenge (ANOVA, *p* = 0.06). All GeMI-Vax groups had similar levels of anti-*E. coli* vector background responses by time of challenge. To characterize whether the *Pb*CSP-specific antibodies recognize native antigen, IFAs with *P. berghei* sporozoites were performed (Figure 5). Only sera from mice immunized with either OM-*Pb*CSP or the recombinant *Pb*CSP had IFA-reactive antibodies. The mean endpoint titer of the OM-*Pb*CSP was 1:64,000, while the mean endpoint titer of the recombinant *Pb*CSP was 1:125,000. Sera from other GeMI-Vax groups failed to react with sporozoites above the background level of the empty GeMI-Vax group. Lack of reactivity to sporozoites could be due to: (a) GeMI-Vax expressing the *Pb*CSP-induced epitope specificities not present or accessible in the native CSP on sporozoites or (b) the concentration or avidity of the antibodies was too low to detect binding or (c) the inability of the intracellular *Pb*CSP delivery to significantly mount humoral responses at the doses of antigen delivered. Avidity measurements were performed using the chaotropic agent NaSCN in ELISAs to compare the OM-*Pb*CSP and soluble protein *Pb*CSP/ISA 720 responses. OM-*Pb*CSP-induced antibody responses with enhanced avidity compared to the conventional antigen/adjuvant approach (ED_50_ NaSCN (mol/L) for OM-*Pb*CSP and *Pb*CSP/ISA 720, 2.0 versus 1.0, respectively).

**Figure 6:**
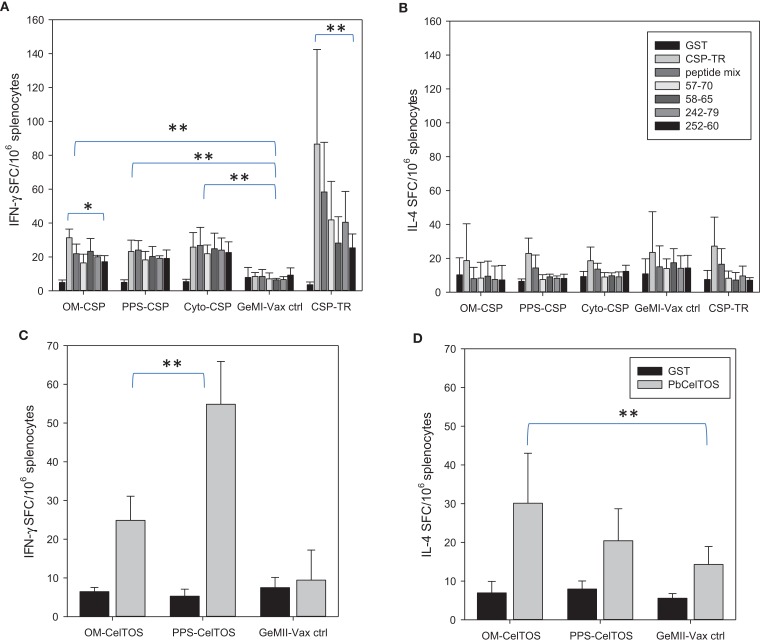
***E. coli* GeMI-Vax induce Th1 but not Th2 responses**. GeMI-Vax constructs (indicated on the *X*-axis) were tested for their ability to mount antigen-specific IFN-γ responses **(A,C)**, and IL-4 responses **(B,D)** after antigen-specific stimulation. **(A,B)** Splenocytes stimulated *ex vivo* with either recombinant *Pb*CSP (10 μg/mL) or *Pb*CSP-derived peptides (peptide mix = pool of all four peptides, peptides CS 57-70 and CS 242-279 represent I-A^d^ restricted CD4^+^ epitopes, peptides CS 58-65 and CS 252-60 represents H2K^d^-restricted CD8^+^ epitopes). GST = negative control. **(C,D)** Splenocytes stimulated *ex vivo* with either recombinant *Pb*CelTOS or GST protein (30 μg/mL). Data are expressed as the mean and standard error of the mean (SEM) number of spot-forming cells (SFC) from *n* = 5 mice/group. Asterisk indicates statistical significance (Students *T*-test): *p < 0.05, **p < 0.001.

To determine the effect of antigen localization on the induction of cellular responses, spleens from immunized mice were stimulated *ex vivo* with recombinant *Pb*CSP as well as *Pb*CSP-derived peptides representing immunodominant CD4 or CD8 epitopes. T cell responses were measured by the number of IFN-γ and IL-4 producing cells responding to antigen stimulation in ELISpot assays (Figures 6A,B). The responses to the recombinant protein and peptides for the r*Pb*CSP/ISA 720 group were significantly higher than for any GeMI-Vax group (*p* < 0.03, *p* < 0.001, respectively, Student’s *T*-test). Interestingly, the magnitude of the T cell response did not parallel the magnitude of the antibody response since both the PPS-*Pb*CSP and Cyto-*Pb*CSP groups had similar numbers of IFN-γ spot-forming cells (SFC) as the OM-*Pb*CSP group. The IFN-γ responses measured in the GeMI-Vax groups were significantly higher than those observed in the empty GeMI-Vax (control group) demonstrating antigen-specificity (*p* < 0.001, ANOVA). In contrast to the antibody responses, the differences in antigen localization and doses delivered by the different bacterial vectors did not appear to affect the overall magnitude of the cellular responses. Neither the GeMI-Vax groups nor the *Pb*CSP/ISA 720 group mounted a significant Th2 type cellular response, as indicated by the overall lack of IL-4 production (Figure 6B).

To further explore the role of cellular localization and the modulation of immune responses, similar constructs to those describe for *Pb*CSP were generated for expressing the *Pb*CelTOS antigen (i.e., OM-*Pb*CelTOS and PPS-*Pb*CelTOS). CelTOS is a secreted, micronemal protein expressed from salivary gland sporozoites through early liver cell infection stages. Preclinical experiments utilizing recombinant protein revealed that CelTOS-based immunity is highly conserved and results in protection against sporozoite challenge in a homologous (44) and a heterologous (45) challenge model and that the protection is dependent on both cellular and humoral immunity (44). The observed cross-species protection is a unique feature of the CelTOS antigen and allows for evaluation of *P. falciparum* CelTOS in the *P. berghei* murine model. Unlike for OM-*Pb*CSP, mice immunized with OM-*Pb*CelTOS did not induce significant levels of antibodies above background (data not shown); however, a significant cellular response that was skewed toward Th1 was evident (Figures 6C,D). In this case, localization of CelTOS to the surface or the PPS yielded IFN-γ responses that were significantly different (*p* < 0.001, *T*-test). Only the OM-*Pb*CelTOS construct induced IL-4 responses that were significantly different from the empty GeMI-Vax control (*p* < 0.01, *T*-test).

To evaluate the *in vivo* efficacy, BALB/c mice were challenged by injecting 4,000 salivary gland *P. berghei* sporozoites subcutaneously (SQ). We have previously shown that the SQ route better mimics the natural infection route than does the intravenous route (IV) by engaging humoral immune mechanisms (47). Mice that remained aparasitemic on day 14 after challenge were considered sterilely protected (Table 2). Sterile protection was observed in mice immunized with the OM-*Pb*CSP, while PPS- and Cyto-*Pb*CSP were only able to induce partial protection. Some mice were re-challenged 10 weeks after the initial challenge to determine (1) whether protection was long-lasting, and (2) whether the exposure to the parasites during the first challenge edited the immune response (48). Only mice immunized with OM-*Pb*CSP achieved complete protection (7/7 mice) upon re-challenge (Table 2). Although low numbers of animals were re-challenged, these results suggest the GeMI-Vax platform is capable of inducing long-lasting immunity.

**Table 2:** **GeMI-Vax induce long-lasting protective immunity that varies by sub-cellular localization**.

Immunization group	First challenge			Re-challenge^d^		
	Protected/total	Efficacy (%)^a^	*p*-Value^b^	Protected/total	Efficacy (%)^a^	*p-*Value^b^
OM-*Pb*CSP^d^	27/35	68	<0.0001	7/7	100	0.0002
PPS-*Pb*CSP^d^	17/25	55	0.02	3/6	50	0.06
Cyto-*Pb*CSP	4/10	14	0.5	3/4	75	0.02
r*Pb*CSP/ISA 720^d^	24/30	76	<0.0001	7/8	88	0.0007
ISA 720^d^	5/29	–	–	ND	–	–
Empty GeMI-Vax^d^	10/34	–	–	ND	–	–
Naïve^c^	ND	–	–	0/8	–	–
OM-*Pb*CelTOS	5/10	29	0.144	ND	–	–
PPS-*Pb*CelTOS	7/10	58	0.027	ND	–	–

*^a^Efficacy (%) = {1 − [(Exp_infected_/Exp_total_)/(Cont_infected_/cont_total_)]} × 100*.

*^b^Significance was determined by using Fisher’s Exact Test*.

*^c^Naïve mice served as infectivity controls for the re-challenge experiment*.

*^d^Protection results from three independent experiments. Only mice from the first protection study were re-challenged*.

*ND = not done*.

### Translation to clinically relevant *Shigella flexneri* 15G-GeMI-Vax expressing malarial antigens

After the initial success using *E. coli*, the GeMI-Vax platform was adapted to an *asd* mutant of *S. flexneri 2a (*15G strain). The rationale for switching to *Shigella* was based on safety concerns related to immunization with a commensal *E. coli* present in the host gut. Attenuated derivatives of the *S. flexneri* 2a have previously been tested as oral vaccines to prevent shigellosis (30, 53, 54) and used in animal studies as potential multivalent mucosal vaccine candidates for delivery of bacterial antigens and eukaryotic genes (55). An *asd* mutant of *Shigella* is unable to grow in the absence of DAP, an essential peptidoglycan component of the bacterial cell wall. Since mammalian cells lack DAP, *Shigella asd* mutants can readily invade epithelial cells but are unable to replicate thus rendering them highly attenuated. Facile transition from *E. coli* to *Shigella* was achieved due to their genetic similarities thus allowing identical approaches for establishing the GeMI-Vax *Shigella* concept. The malaria targets were derived from the lethal *Plasmodium* spp., *P. falciparum*, and were engineered for cellular localization as described for the *P. berghei* CSP and CelTOS. Based on results from *E. coli* GeMI-Vax, the *Pf*CSP was targeted to the OM while the CelTOS antigen was targeted to the PPS of *Shigella*. Because of its native surface localization, the objective for the OM-*Pf*CSP was to achieve strong antibody responses while the intracellular PPS-*Pf*CelTOS was intended to induce primarily T cell responses. Since in these experiments *Shigella* GeMI-Vax was delivered by parenteral routes the immune responses were focused on the malaria antigens and not the *shigella* delivery vehicle.

**Figure 7:**
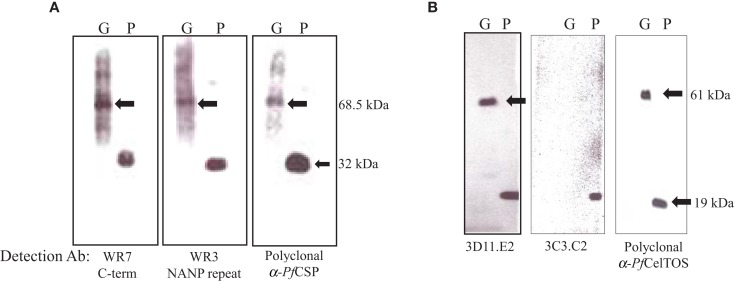
**Analysis of expression levels of malarial antigens from the different sub-cellular compartments on *Shigella* GeMI-Vax**. Lysates from GeMI-Vax expressing OM-*Pf*CSP **(A)** or PPS-*Pf*CelTOS **(B)** were probed with antigen-specific mAbs and polyclonal Abs: clone WR7 = *Pf*CSP C-terminus specific mAb; clone WR3 = *Pf*CSP repeat region-specific mAb, polyclonal = serum pooled from mice immunized with recombinant *Pf*CSP; 3D11.E2 = *Pf*CelTOS-specific, conformation-dependent mAb; 3C3.C2 = *Pf*CelTOS-specific, conformation-independent mAb; polyclonal = serum pool from rabbits immunized with recombinant *Pf*CelTOS/ISA 720. Arrows indicate fusion protein in molecular mass. G is GeMI-Vax lysate; P is recombinant protein. Molecular weight of the r*Pf*CSP is 34 kDa and the r*Pf*CelTOS is 19.1 kDa.

Expression levels of the *S. flexneri* 15G encoding either OM-*Pf*CSP or PPS-*Pf*CelTOS were confirmed by Western blotting with antigen-specific mAbs (Figure 7). OM-*Pf*CSP was detected by polyclonal mouse antisera as well as mAbs specific for the C-terminus (clone WR7) or the central repeat, NANP region (clone WR3) (Figure 7A) of the *P. falciparum* CSP. Both the C-terminal and the repeat region-specific mAbs recognized the OM-*Pb*CSP fusion protein (68.5 kDa) and the recombinant full-length protein, r*Pf*CSP (∼32 kDa). For immunizations, the amount of OM-*Pf*CSP delivered was estimated to be 0.05 μg (low dose) and 5 μg (high dose) per dose based on immunoreactivities on Western blots. Expression of PPS-*Pf*CelTOS was confirmed by Western blots probed with CelTOS specific mAbs and polyclonal antibodies (Figure 7B). The fusion protein was confirmed to have a size of ∼61 kDa. Fine specificity of the *Pf*CelTOS-specific mAbs indicate that anti-*Pf*CelTOS mAb 3C3.C2 binds primarily to linear C-terminal epitopes while mAb 3D11.E2 binds primarily to the recombinant *Pf*CelTOS protein indicating specificity for a conformational epitope. The lack of immunoreactivity by the mAb 3C3.C2 to the PPS-*Pf*CelTOS protein suggests that the binding epitope for this mAb may not be accessible due to folding constraints. Both mAbs 3C3.C2 and 3D11.E2 and the polyclonal immune sera recognized the reference protein, r*Pf*CelTOS (19 kDa). Based on Western blot probing the amount of PPS-*Pf*CelTOS delivered during immunizations was estimated to be ∼0.01 μg (low dose) and 1 μg (high dose) per dose.

### Induction of PfCelTOS or PfCSP-specific antibody responses by *Shigella* GeMI-Vax depends on antigen localization

**Figure 8:**
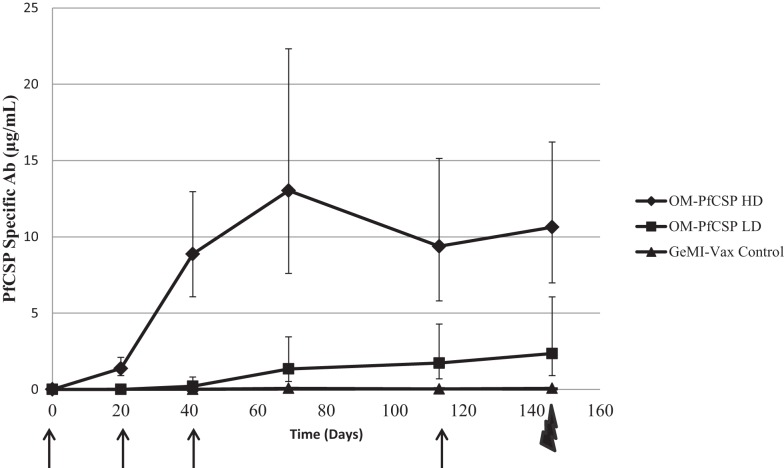
**Kinetics of the humoral immune responses induced by *Shigella* GeMI-Vax**. Sera from mice immunized with OM-*Pf*CSP HD (high dose) and OM-*Pf*CSP LD (low dose) were collected prior to, 3 weeks after each immunization and 1 day prior to the challenge and were analyzed using a quantitative ELISA. Data are expressed as the geometric mean *Pf*CSP-specific antibody concentration (μg/mL) and the error bars represent the 95% confidence interval of *n* = 10 mice/group. Arrows indicate the days of immunizations, Day-1, 20, 41, and 113, and the bolt indicates time of challenge. Mice were immunized with either high dose = 1.7 × 10^9^ cells or low dose = 1.7 × 10^7^ number of cells. At each time point following the primary immunization, *Pf*CSP-specific antibody responses for the OM-*Pf*CSP HD group were significantly higher than for the OM-*Pf*CSP LD group (ANOVA, *p* < 0.05).

The immunogenicity of GeMI-Vax expressing *Pf*CelTOS or *Pf*CSP was evaluated by immunizing mice with a high dose (1 μg) or low dose (0.01 μg) of PPS-*Pf*CelTOS or high dose (5 μg) or low dose (0.05 μg) of OM-*Pf*CSP. The doses were selected based on the initial experiments performed with *E. coli* where 1.7 × 10^9^ cells/mouse/dose was the highest tolerated dose. The kinetics of the antibody response in the immunized mice confirmed the earlier observation that targeting the antigen to the OM results in more robust humoral responses (Figure 8). Due to issues related to the availability of *P. berghei Pf* CSP-transgenic sporozoites for challenge it was necessary to include a fourth immunization of the OM-*Pf*CSP GeMI-Vax. Delivering a higher dose of GeMI-Vax cells yielded statistically significant higher antigen-specific antibody responses at all time points (ANOVA, *p* < 0.05). Based on antibody kinetics, the fourth immunization did not significantly boost the response in the group receiving the high dose of OM-*Pf*CSP. In contrast, the PPS-*Pf*CelTOS groups did not show any significant antigen-specific antibodies above the background empty GeMI-Vax control cells (data not shown). IFA-results using *P. falciparum* sporozoites paralleled and confirmed the results from the ELISA in that immunization with OM-*Pf*CSP led to high titers of CSP antibodies that are sporozoite-reactive, while the titers induced by the PPS-*Pf*CelTOS were negligible (data not shown).

### T cell responses differed by targeting to cellular components

**Figure 9:**
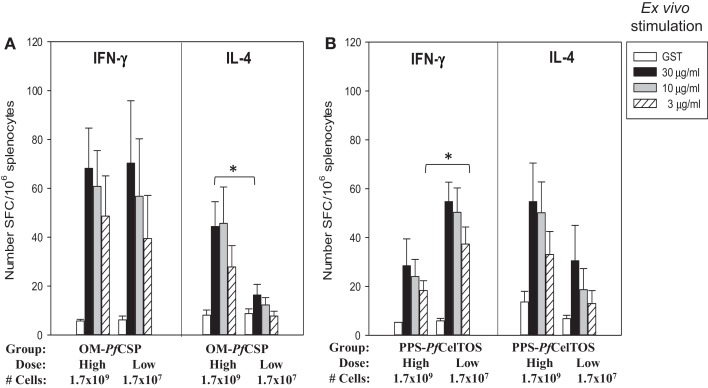
***Shigella* GeMI-Vax-induced a balanced T cell response that is dose-independent**. Splenocytes from mice immunized with either OM-*Pf*CSP **(A)** or PPS-*Pf*CelTOS **(B)** were stimulated *ex vivo* with the respective recombinant protein (either r*Pf*CSP or r*Pf*CelTOS) or GST (negative control). Data are expressed as the mean and standard error from the mean (SEM) number of spot-forming cells (SFC) from *n* = 5 mice/group. Mice were immunized with either High dose = 1.7 × 10^9^ or Low dose = 1.7 × 10^7^ number of cells. Asterisk indicates statistical significance (Students *T*-test; *p < 0.05).

Immunization with OM-*Pf*CSP induced *Pf*CSP antigen-specific IFN-γ and IL-4 producing T cells with an apparent bias toward Th1-type responses compared to stimulations with irrelevant protein (GST as negative control) (Figure 9A). In contrast to the IFN-γ responses, the magnitude of the IL-4 responses was dependent on the immunization dose, however, only splenocytes stimulated with 30 μg/mL *Pf*CSP yielding a significant difference between the two dosage groups (Student’s *t*-test; *p* < 0.05). Immunization with the PPS-*Pf*CelTOS induced a relatively more balanced T cell response that did not significantly change as a function of the immunization dose, except for the low dose PPS-*Pf*CelTOS splenocytes stimulated with 3 μg/mL of recombinant CelTOS, which were significantly higher than high dose (Students *t*-test; *p* < 0.05) (Figure 9B). The ability of the PPS-construct to induce antigen-specific T cells while failing to induce significant antibodies supports the earlier finding that localization to the periplasm leads to predominantly cellular responses.

### *Shigella* GeMI-Vax cells induced protective immunity mediated by PfCelTOS in the periplasm

To evaluate the protective efficacy of the PPS-*Pf*CelTOS, mice were challenged with wild-type *P. berghei* parasites (45) while the OM-*Pf*CSP mice were challenged with *Pf*CSP-transgenic *P. berghei* parasites (46). A summary of the *Shigella* GeMI-Vax-induced protection is shown in Table 3. The level of protection induced by PPS-*Pf*CelTOS was comparable to previously reported results using 10 μg *Pf*CelTOS/Montanide ISA-720 (45). The effective antigen dose delivered by *Shigella* PPS-*Pf*CelTOS was 10-fold lower than for soluble protein/adjuvant. When equivalent low doses of recombinant protein were delivered as soluble protein/adjuvant, no protection was observed (45), suggesting that the PPS-*Pf*CelTOS GeMI-Vax approach is antigen dose sparing. Although the high dose of *S. flexneri* GeMI-Vax OM-*Pf*CSP also induced a dose-dependent, antigen-specific antibody response, no significant protection was observed in contrast to the observations with the OM-*Pb*CSP in *E. coli*.

**Table 3:** **Immunization with high dose PPS-PfCelTOS protects mice against challenge with wild-type *P. berghei* sporozoites**.

Immunization group	Parasite challenge	Dose^a^, #Cells	Protected/total	Efficacy (%)^b^	*p*-Value^c^
OM-*Pf*CSP	*Pf*CSP-transgenic (IV route)	High, 1.7 × 10^9^	2/9	13.5	0.46
		Low, 1.7 × 10^7^	4/10	33	0.15
Empty GeMI-Vax		High, 1.7 × 10^9^	1/10	–	–
Saline		–	0/10	–	–
PPS-*Pf*CelTOS	*P. berghei* WT (SC route)	High, 1.7 × 10^9^	6/10	55	0.04
		Low, 1.7 × 10^7^	4/10	32.5	0.18
Empty GeMI-Vax		High, 1.7 × 10^9^	1/9	–	–

*^a^High dose = 1.7 × 10^9^ cells; low dose = 1.7 × 10^7^ cells*.

*^b^Efficacy (%) = {1 − [(Exp_infected_/Exp_total_)/(Cont_infected_/Cont_total_)]} × 100*.

*^c^Significance was determined by using Fisher’s Exact test*.

## Discussion

New and improved methods for the simultaneous presentation of pathogenic antigens and immune stimulation are needed for the development of efficacious vaccines. Platforms that present antigen to the host immune system in a particulate manner can mimic the structure of a natural pathogen leading to improved immunogenicity. GeMI-Vax are intact, Gram-negative bacteria, that are inactivated through genetic means and provide a delivery platform for presenting xenogeneic expressed target antigens with intrinsic danger signals to the host immune system. Although immunostimulatory, and from a safety standpoint, the endotoxin constituent of Gram-negative OMs does not limit their use as vaccine vectors primarily due to their minimal toxicity when presented as cell-associated lipopolysaccharides compared to the free-soluble forms (56).

In this study, GeMI-Vax served to evaluate the induction of antigen-specific humoral and cellular immune responses by expressing malaria proteins to different sub-cellular localizations. Localization of the *Pb*CSP in *E. coli* GeMI-Vax to different cellular compartments induced distinct immune responses. *Pb*CSP fused to the OM induced both humoral and Th1 cellular responses that induced sterile protection against homologous sporozoite challenge. Immunostaining of OM-*Pb*CSP GeMI-Vax verified the surface localization and revealed a punctate and circumferential staining pattern that mimicked the authentic conformational display of CSP on sporozoites. Interestingly, none of the intracellular *Pb*CSP localizations whether to the PPS or to the cytosol, induced high levels of *Pb*CSP-specific antibodies. However, sub-cellular localizations of *Pb*CSP led to the induction of IFN-γ responses by both CD4 and CD8 T cells, recognizing peptide epitopes throughout *Pb*CSP (Figure 6). In contrast to *Pb*CSP, OM localization of the *Pb*CelTOS did not lead to pronounced levels of antibody. These results point to both cellular localization and the nature of the target antigen in influencing the induced immunity. Thus the key immunological findings are (1) protection was highest in the groups that mounted significant antibody responses, (2) *Pb*CSP-antigen delivered by GeMI-Vax from different sub-cellular localizations does not influence the magnitude of the IFN-γ responses; and (3) none of the *Pb*CSP-GeMI-Vax constructs induced significant *Pb*CSP-specific IL-4 responses. These findings are consistent with other studies where *Pb*CSP-specific antibodies were shown to play an important role in protection against a homologous sporozoite challenge (48). In the current study, the longevity of the protective response and its robustness against parasitic editing was measured by re-challenging the protected mice 10 weeks after the first challenge (Table 2). Only the OM-*Pb*CSP immunized mice which notably had the highest antibody response and a concomitant Th1-type cellular response were completely protected. Currently, it is unclear whether the surface presentation on bacteria led to extended persistence of the antigen on follicular dendritic cells as described for other targeted vaccines (48). Another explanation for the sustained protection could be an increase in the precursor frequency of high-avidity B cells induced by lower antigen doses in the context of a potent activator of the innate immune system, a hypothesis supported by the finding that immune sera induced by OM-*Pb*CSP GeMI-Vax had higher avidities than did the soluble protein/adjuvant. Thus presentation of *Pb*CSP on the bacterial surface likely resembled the natural surface display on sporozoites and the adjuvanticity of the bacterium led to robust humoral responses despite the lower antigen dose. Concomitantly, surface display of PPS-*Pb*CelTOS did not generate significant levels of antibodies and the protection seen was primarily due to a potent Th1-type IFN-γ T cell response. The present study emphasizes that the nature of the antigen; and both the quantity and quality of vaccine-induced immune responses, have a role in the potency of this vaccine platform.

The efficacy of *Shigella* GeMI-Vax OM-*Pf*CSP differed from the results obtained in *E. coli* since the OM-*Pf*CSP did not lead to significant protection against sporozoite challenge. One difference between the two experiments was the challenge route: the *Pb*CSP *E. coli* GeMI-Vax construct were evaluated using the subcutaneous injection with wild-type *P. berghei* sporozoites which more closely mimics the natural challenge route (mosquito bite) (47). In contrast, the *Pf*CSP *Shigella* GeMI-Vax constructs were evaluated using intravenous injection of *Pf*CSP-transgenic *P. berghei* sporozoites. By injecting sporozoites directly IV, effector antibodies may have had little chance to contribute to protection (57). In this case, the partial protection may be attributed solely to the cellular responses induced by this vaccine. While the periplasmic location of the *Pb*CSP did not induce statistically significant protective immunity, the opposite was seen with *Pf*CelTOS localized to the PPS. These findings further confirm that the protection is not solely a function of the sub-cellular localization but that (a) the nature of the antigen and (b) the antigen-specific dosage levels may contribute to protection.

These results reveal that GeMI-Vax expressing the two major pre-erythrocytic targets, CSP or CelTOS, can elicit protective efficacy against homologous sporozoite challenge in murine models. In comparison with other vaccine technologies, the GeMI-Vax platform has several advantages: (a) ease and low cost of manufacturing; (b) highly stable final lyophilized product well suited for cold chain-free transport to remote field sites; (c) self-adjuvanting; (d) induces high-avidity antibodies known to be more efficacious in mediating protection; (e) triggers distinct immune responses by the choice of cellular localization of the target antigen; (f) integration with Gram-negative human pathogens such as *Shigella* GeMI-Vax holds the possibility for a dual-use vaccine to mediate protection against shigellosis and xenogeneic antigen(s) expressed by the bacteria. Finally, based on practical issues, GeMI-Vax may be suitable for use in the early immunological characterization of novel target antigens identified from high throughput antigen discovery projects. Using relatively standard molecular engineering, novel targets can be expressed and delivered to assess their immune potential in murine models without time consuming protein purification and process development. GeMI-Vax is also suitable for high density display of target antigens and can be easily integrated into a multi-epitope, multi-stage malaria vaccine system.

## Disclaimer

The authors’ views are private and are not to be construed as official policy of the Department of Defense or the U.S. Army. Research was conducted in compliance with the Animal Welfare Act and other federal statutes and regulations relating to animals and experiments involving animals and adheres to principles stated in the *Guide for the Care and Use of Laboratory Animals*, NRC Publication, 1996 edition.

## Conflict of Interest Statement

The authors declare that the research was conducted in the absence of any commercial or financial relationships that could be construed as a potential conflict of interest.
